# Misfit Strain Relaxation of Ferroelectric PbTiO_3_/LaAlO_3_ (111) Thin Film System

**DOI:** 10.1038/srep35172

**Published:** 2016-10-11

**Authors:** Y. B. Xu, Y. L. Tang, Y. L. Zhu, Y. Liu, S. Li, S. R. Zhang, X. L. Ma

**Affiliations:** 1 Shenyang National Laboratory for Materials Science, Institute of Metal Research, Chinese Academy of Sciences, 72 Wenhua Road, 110016 Shenyang, China

## Abstract

Ferroelectric thin films grown on high index substrates show unusual structural and switching dynamics due to their special strain states. Understanding the misfit relaxation behavior is crucial to facilitate the high index thin film growth with improved quality. In this paper, ferroelectric PbTiO_3_ thin films were grown on LaAlO_3_ (111) substrates by pulsed laser deposition technique. The microstructures were investigated by combinations of conventional and aberration-corrected transmission electron microscopy. Diffraction contrast analysis and high resolution imaging reveal that high density interfacial dislocations were distributed at the interfaces. These dislocations have mixed character with Burgers vectors of a <110> and line directions of <112>. The edge components of the dislocations, with the Burgers vectors parallel to the interface, accommodate the lattice mismatch and are the main contributor to the misfit relaxation of this system. The formation mechanism of these dislocations is proposed and discussed to elucidate the novel mismatch relaxation behavior of <111> oriented perovskite films.

High-index ABO_3_ perovskite oxide thin films have attracted much attention recently because of their unusual characteristics in contrast to conventional low-index ABO_3_ films^1^,^2^. It was found that (111)-oriented PbZr_0.2_Ti_0.8_O_3_ showed higher volume fraction of minority domains and line density of 90° domain walls than (001)- and (101)-oriented films, which could make an enormous difference in ferroelectric susceptibility and ferroelectric switching process^3^,^4^. Moreover, the promised novel physical phenomena, which could not be observed in low-index perovskite oxide films, have accelerated the research on high-index perovskite oxide superlattices^5^. Specially, the theory predicted that exotic topological phases (e.g., Dirac half-semimetal phase, quantum anomalous Hall insulator phase, or ferromagnetic nematic phase) could be modulated by the strength of electron-electron correlations along the [111] crystallographic direction^6^. Most recently, Middey *et al*.^7^ realized an artificial graphene-like Mott crystal with magnetic d^7^ electrons by devising bilayers of the rare-earth nickelate NdNiO_3_ along the pseudocubic [111] direction. In multiferroic materials, Chu *et al*.^8^ reported that the domain variants can be effectively controlled in BiFeO_3_ thin film by using substrate orientations as a critical control parameter. Therefore, high-index substrates could not only play an important role in regulating the microstructures of high-index films, but also are important in controlling and obtaining intriguing properties of perovskite oxide thin films.

Unfortunately, a crucial challenge in the implementation of both the theoretical and experimental proposals for those high-index perovskite heterostructures is the difficulty for preparing thin films with high quality along [111] direction. There are many factors to hinder this process. First, high index surfaces involving polar discontinuities tend to increase surface energies for ABO_3_ perovskite oxides in general which will be an enormous challenge for the growth of epitaxial high-index ABO_3_ films^9^. In addition, such polar discontinuity has a huge electrostatic energy cost and thus triggers a series of screening mechanisms that yield the accumulation of free charge at the interface^10^. The polar discontinuities are common for substrates such as SrTiO_3_ (or LaAlO_3_) consist of alternating SrO_3_^4−^and Ti^4+^(or LaO_3_^3−^and Al^3+^) charged planes stacked along the [111] direction. This electrostatic dipole moment normal to the surface yields a divergent surface potentials when the thickness of the films increases, making an unreconstructed (111) surface quite unstable. Next, the epitaxial thin film growth along these highly polar directions may accelerate the occurrence of complex surface reconstructions that act to compensate for the polar mismatch, which will prevent the first (few) monolayers of the films from coherent growth^11^,^12^. Although the epitaxial growth of (111)-oriented ABO_3_ heterostructures was recently reported^11^, it is noted that atomically smooth surfaces and interfaces could not be obtained unless a screening buffer layer was introduced between the substrate and the film. To further understand the film growth with high quality, it is necessary to investigate the microstructures and misfit strain behavior in high-index films.

In addition, owing to the strong coupling between strain and ferroelectricity, misfit strains and defect structures can make an enormous impact on the microstructures and properties of displacement ferroelectrics^13^,^14^. However, as a typical ferroelectric material, studies on the growth of PbTiO_3_ (PTO) thin films on (111)-oriented substrates have been inadequate, even though these tetragonal ferroelectric films grown on such (111) substrates would exhibit novel physical properties different from (001) and (110)-oriented ones^3^,^4^.

In this paper, transmission electron microscopy (TEM) and aberration-corrected scanning transmission electron microscopy (STEM), which could provide resolution to the sub-angstrom level and identify different atom columns directly, were used for investigating interfacial structures and strain/defects in PTO (111) films^15^,^16^. These films were grown epitaxially on LaAlO_3_ (111) (LAO (111)) substrates by pulsed laser deposition (PLD) technique. Misfit dislocations with Burgers vectors of a <110> and line directions of <112> were observed along the interface, which constitute the major interfacial defects in these films. Moreover, the mechanism of relaxing misfit strain between PTO and LAO were discussed. Particularly, the formation mechanism of these novel misfit dislocations is proposed and discussed as well.

## Results

### General information

It is well known that bulk LAO is rhombohedral with the lattice parameters of a_rh_ = 5.63 Å and space group of *R-3c* at room temperature^17^. For the sake of convenience, LAO is generally described in terms of a pseudocubic structure with a lattice parameter of a_p_ = 3.79 Å. In the following, pseudocubic indices will be used and indicated by the subscript “p.” Above Curie temperature, the PTO crystal shows a simple cubic structure with the space group of *Pm-3m* and the lattice parameter of a = 3.95 Å. On cooling through the Curie temperature, the symmetry of the PTO crystal decrease to a tetragonal structure which belongs to the *P4mm* space group with the lattice parameters of a = 3.89 Å, c = 4.14 Å^18^. In order to exactly learn the misfit strain between the films and the substrates, it is necessary to compare the corresponding interplanar spacings rather than the interatomic distances especially for high-index orientations. The pseudocubic LAO (111) surface has two in-plane orthogonal crystal axes: <110> and <112>; the corresponding lattice mismatches lie in between the planes of LAO {110} and PTO {110}, LAO {112} and PTO {112}, which is different from the situation grown on the commonly used LAO (001) where two directions are equivalent. In the pseudocubic LAO substrate, d_p111_ = 2.19 Å, d_p110_ = d_p101_ = d_p011_ = 2.68Å, and d_p112_ = 1.55 Å. The corresponding interplanar spacings in the tetragonal PTO are d_111_ = 2.29 Å, d_110_ = 2.75 Å, d_101_ = d_011_ = 2.83 Å, d_112_ = 1.65 Å, and d_121_ = d_211_ = 1.60 Å. It turns out that the interplanar spacings of PTO are comparable to the corresponding interplanar spacings of LAO_P_. The mismatch *f* is calculated to be −2.58% for d_p110_ and d_110_, −3.17% for d_p112_ and d_121_/d_211_, −6.25% for d_p112_ and d_112_, and −5.44% for d_p110_ and d_101_/d_011_, respectively. It is thus determined that the as-grown films are under compressive and suffer an anisotropic in-plane strains coming from low symmetry surfaces of LAO substrate.

### Conventional transmission electron microscopy

Conventional TEM imaging was performed on Tecnai G2 F30 TEM for acquiring fundamental information of PTO thin films. TEM investigations indicate that the interfacial structures of the PTO/LAO (111) systems are complicated and very different from low-index interfaces.

**Figure 1:**
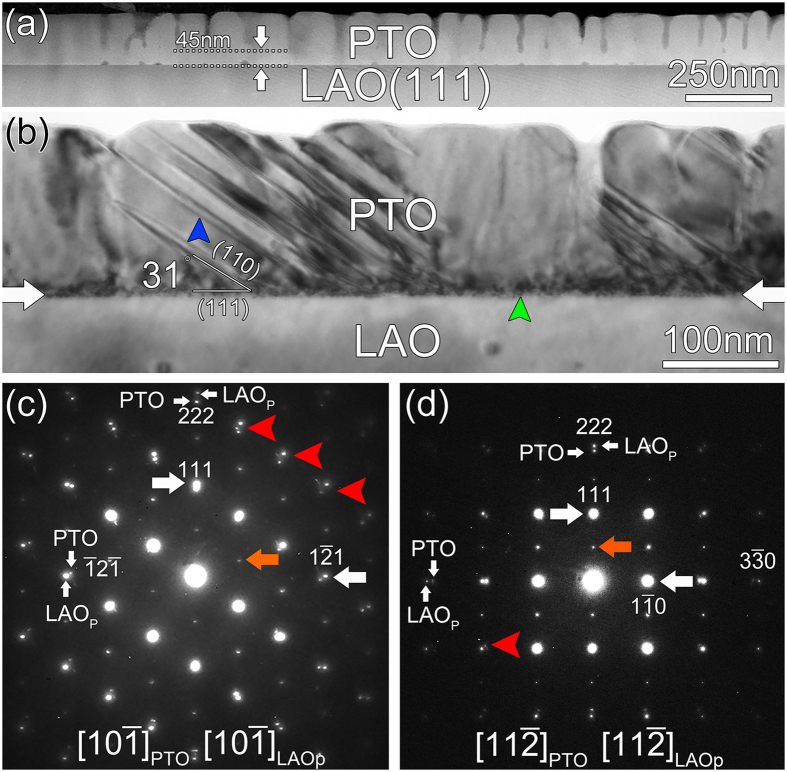
(**a**) Low-magnification cross-sectional HAADF images showing the morphology of PTO thin films grown on the LAO substrate. (**b**) Cross-sectional bright-field image of the PTO/LAO heterostructure showing the accumulation of a high density of defects near the interface. The interface is marked by a pair of white arrows. (**c**) Composite EDPs of (**c**) [10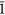]_f_ and (**d**) [11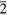]_f_. Spot splitting due to the different lattice parameters of PTO and LAO_P_ can be observed. Subscripts s and f denote the LAO substrate and PTO films, respectively.

Figure 1(a) is a low-magnification HAADF image showing an overview of the PTO thin films grown on LAO substrates. The thickness of the whole film is about 160 nm. It is noted that the film exhibits a two-layered structure: a continuous layer with 45nm thickness directly grown on the substrate, and a nanostructured layer on top of the continuous layer. The nanostructured layer is columnar-like with an average width of 85 nm for each column. When TEM bright-field imaging was performed on conventional TEM, the contrast in the film varies dramatically as shown in Fig. 1(b). From this image, it is noted that a high density of black dot-like defects is accumulated near the interface as denoted by a green arrow. In addition, some stripe-like contrast can be observed in the film as indicated by a blue arrow. They form about 31° angle with the interface, which is close to the angel between (110) plane and (111) plane. It is known that ferroelectric domains can be formed in PTO films grown on (111) LAO substrates when PTO experiences phase transition from cubic to tetragonal during the cooling process after film deposition, and the domain wall of (111)-oriented tetragonal PTO is on the (110) plane. So this stripe-like structure should be ferroelectric domain boundaries. But so far it is difficult to confirm the nature of those domains due to their complicated configurations.

Electron diffraction patterns clarify that the as-received PTO/LAO film system possesses well orientation relationships as shown in Fig. 1(c,d) which were taken from the area including the substrate, the continuous layer and the columnar layer, and indexed as [10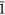] and [11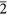] zone axes, respectively. Subscripts f and s denote the PTO films and the LAO substrates, respectively. Orange arrows mark the extra diffraction spots of rhombohedral LAO. With reference to the pseudocubic cells of LAO, a simple cubic-to-cubic orientation relationship can be derived from the electron diffraction patterns. Besides strong diffraction spots from the film and substrate, no extra spots can be observed, indicating that the films are free of secondary phase and no chemical reaction occurs along the interface. In addition, the high-order spots splittings can be identified in Fig. 1(c,d), which are due to the difference in lattice constants of PTO and LAO_P_. The splitting of the spots indicates that mismatch relaxation between the PTO and LAO lattices occurred. Moreover, we can see some extra weak diffraction spots denoted by red arrows which should be from domains (slanted lines in 1b).

**Figure 2:**
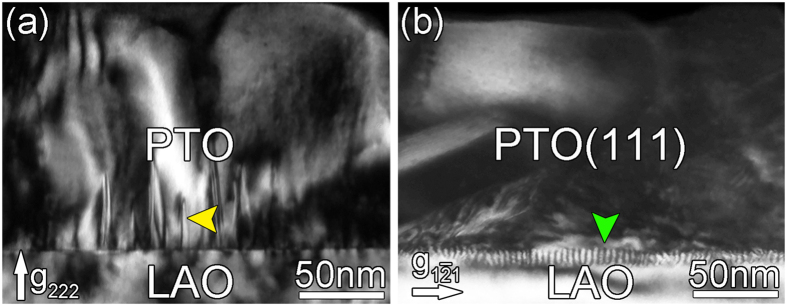
(**a**,**b**) Two-beam dark-field images of cross-sectional PTO/LAO thin films by using (222) and (1
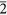
1) reflections, respectively. High density misfit dislocations may accumulate at the interface as denoted by arrow in (**b**).

**Figure 3:**
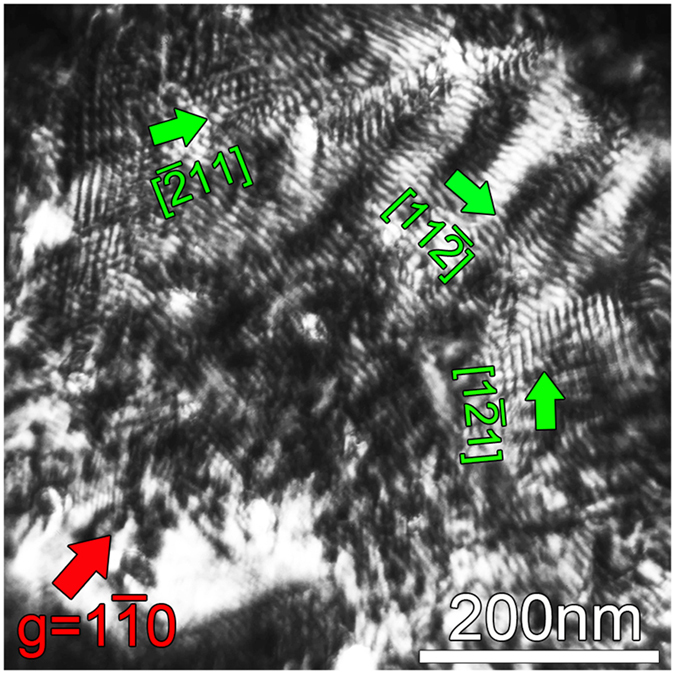
Two-beam dark-field images in plane-view observation using g = 1
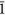
0. Three sets of dislocation lines can be traced along [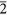
11], [11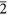] and [1
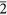
1], respectively.

In order to clarify the high density black dots near the interface, conventional TEM diffraction contrast analysis was carried out. It is found that the contrast changes dramatically associated with the g vectors. Figure 2(a,b) are two-beam dark-field images using different reflections under [10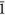] zone axis. The line contrast can be seen in Fig. 2(a) as indicated by yellow arrows when g = 222. They could be planar defect or threading dislocations. Besides, many short lines are seen at the LAO–PTO interfaces as indicated by green arrow in Fig. 2(b) when recorded using (1
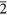
1). It is noteworthy that they are out of contrast in Fig. 2(a) when using the (222) reflections. According to the dislocation contrast extinction criterion: g·b = 0, those short lines may be interfacial dislocations. The Burgers vectors of these dislocations are parallel to the interface. To get more information on the defect characteristics, plane-view observation is necessary. Figure 3 is two-beam dark-field images of a plane-view sample. Three sets of line networks were observed when the imaging condition is under g = 1
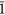
0. The line directions are along [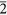
11], [11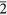] and [1
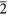
1] of LAO_P_, respectively. Those lines might be misfit dislocation networks related to the lattice mismatch between the film and the substrate. If they were dislocation networks, the spacing (*S*) of the parallel dislocations could be given by:


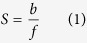


*b* is the magnitude of Burgers vectors of misfit dislocations^19^. For LAO substrate, d_p110_ = d_p101_ = d_p011_ = 2.68Å. For the tetragonal PTO, d_110_ = 2.75Å and d_101_ = d_011_ = 2.83Å. Thus the theoretical dislocation spacings are about 10 nm and 5 nm for d_110_ = 2.75Å and d_101_ = d_011_ = 2.83Å, respectively. Experimentally, the line spacings in Fig. 3 are in the range of 5~10 nm, which thus agrees well with the theoretical values. These observations confirm that the misfit strain between PTO and LAO (111) is mainly relieved by the formation of misfit dislocations and indicate that the dislocation lines probably traced along three equivalent <112> in-plane directions. Note the non-uniformly distributed dislocation networks in Fig. 3 may result from the bending of the plane-view sample or inhomogeneous distribution of strains at the interfaces. The mixed pattern of both misfit dislocations and possible Moiré fringes may result from the variation of diffraction conditions. This bending deformation is common for plane-view TEM samples since they were thinned only from the substrate side.

It is noted that the density of misfit dislocation lines is high because of large misfit strains between PTO and LAO_P_. This kind of misfit dislocation configuration is very different from the ones in previous studies on (001) and (110)-oriented thin film systems^20^,^21^, where misfit dislocations form a rectangular network with line directions of <100>, or a network with line directions of <111> and <100>, respectively. It is usually believed that the misfit dislocations in perovskite-based thin films have the Burgers vectors a <100> and a <110>^20^,^21^,^22^,^23^. The misfit dislocation configuration in the present study is complex and it is difficult to determine the Burgers vectors of the dislocations simply by diffraction contrast analysis. Nevertheless, plane-view low-magnification TEM imaging is a straightforward way to estimate the dislocation density and facilitates further identification of misfit dislocations by high resolution TEM imaging.

### HRSTEM analysis of the misfit dislocations

To accurately determine the Burgers vectors, aberration-corrected HAADF-STEM experiments were performed because of their high spacial resolution and high chemical sensitivity.

**Figure 4:**
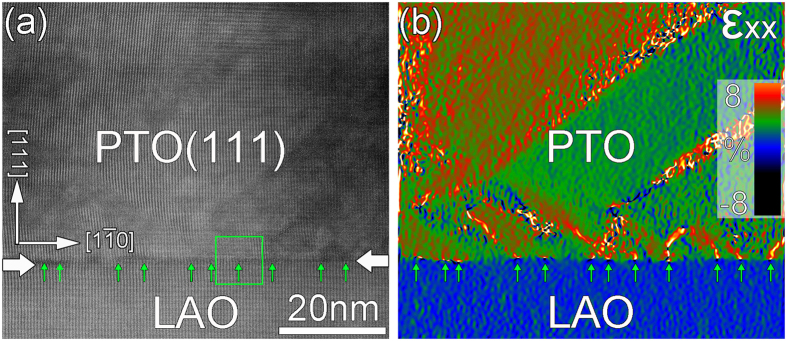
Low magnification HRSTEM image of the PTO thin film on LAO substrates taken along [10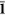] direction. Vertical arrows denote the positions of interfacial dislocations. (**b**) In-plane strain ε_xx_ mapping corresponding to Fig. 4(**a**) showing the strain variations at the interfaces and in the films.

Figure 4(a) is a low-magnification high resolution cross-sectional HAADF-STEM image taken along the [11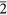] direction of LAO_p_ showing the interface of the PTO/LAO (111) film. An array of misfit dislocations is found nearly periodically distributed along the interface, and the position of each dislocation is denoted by vertical green arrows. The dislocation are spaced about 8~10 nm apart. For showing the locations of dislocations more clearly, the positive strains along the [1
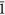
0] direction of PTO was extracted by geometrical phase analysis (GPA) and shown in Fig. 4(b). It is noted that some bright contrast dots denoted by vertical arrows appear at the interface, which may correspond to the positions of dislocations. The large strain variation across the interface implies that the mismatch was largely relaxed by the formation of misfit dislocations. Besides, the clear lines which separate the green and red areas in Fig. 4b might be ferroelastic domain walls, which may cause the sharp contrast of strain states in two sides.

**Figure 5:**
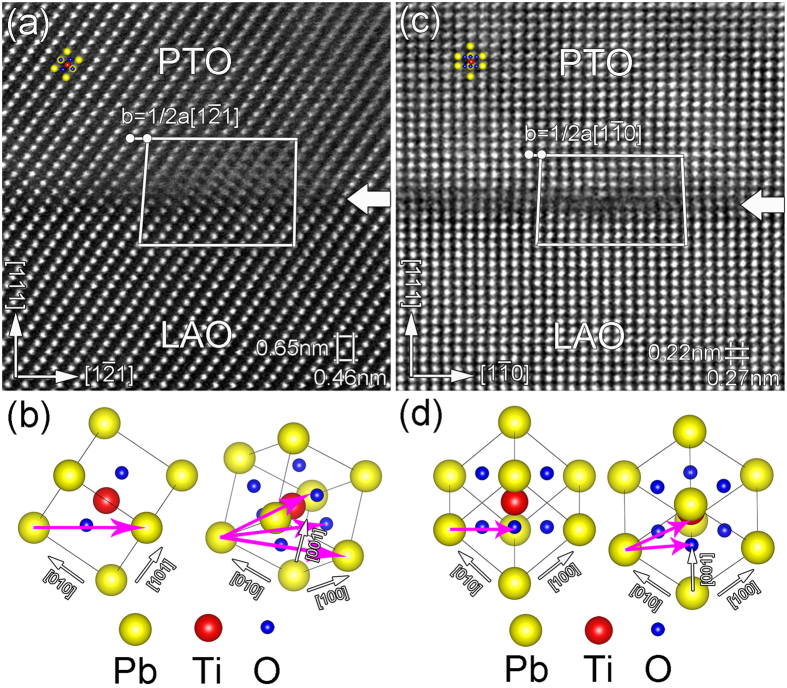
(**a**) HRSTEM image of the area outlined by green box in Fig. 4(a). (**b**) Schematic illustration of the possible Burgers vectors viewed along [10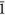] direction. (**c**) The same image as in Fig. 5(**a**) viewed along [11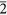] direction. (**d**) Schematic diagram illustrating the possible Burgers vectors viewed along [11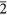]. Combining (**b**) with (**d**), only Burgers vector b = a[0
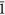
1] is possible.

To display the structural details of misfit dislocations, atomic scale HAADF-STEM images were performed as well. Figure 5(a,b) are the enlarged images of the green box area in Fig. 4(a) taken along [01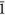] and [11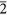] directions of LAO_p_, respectively. As shown in Fig. 5(a), the dislocation core is blurred due to the non-edge-on effect^21^,^24^, i.e. the dislocation line is inclined to the incident electron beam. By drawing a Burgers closure, the projected Burgers vector is determined to be 1/2a[1
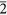
1] in this direction. As mentioned above, misfit dislocations in perovskite-based thin films usually have perfect Burgers vectors <100> and <110>. As schematically illustrated in Fig. 5(b), since the 1/2a[1
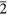
1] projected component is already larger than *a* <100>, the corresponding perfect dislocation are probably a <110> type. To clear this point, it is essential to tilt the sample and measure corresponding projected Burgers vector from another direction. Figure 5(c) is the image taken from [11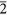] of LAO_p_. In this image, it is noted that the misfit dislocation core looks sharp and no blurring, implying that the dislocation is edge-on and possess [11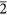] line direction. By drawing a Burgers closure, the Burgers vector is determined to be 1/2a[1
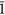
0]. From the schematic diagram in Fig. 5(d), the Burgers vector may be 1/2a[1
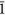
0] or a[0
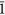
1]. Combined with Fig. 5(a,c), only one perfect Burgers vector can be identified, that is a[0
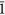
1].

## Discussion

It is well known that the misfit dislocation is an important way to relax strains between film and substrate^22^,^23^. The misfit dislocations in present PTO/LAO (111) thin film were identified to have Burgers vector of a <110> and line directions of <112>. To analyze the misfit relaxation comprehensively, it is reasonable to decompose the perfect dislocation into two components: one is parallel to the line direction and the other is perpendicular to the line direction. Take the [11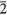] line direction and b = a[0
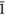
1] as an example, b = a[0
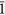
1] can be decomposed according to the following equation:


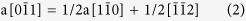


**Figure 6:**
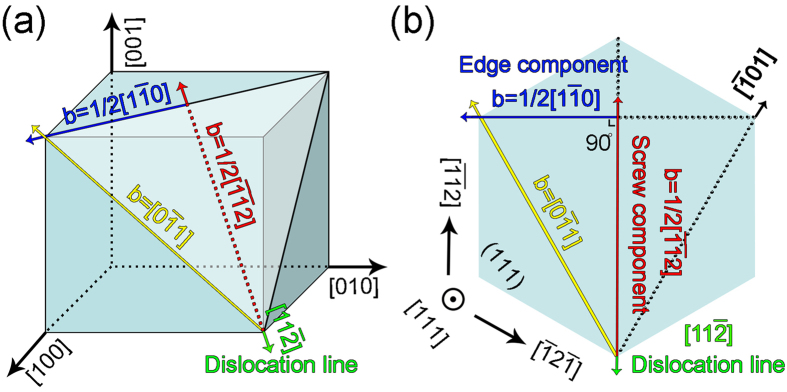
Schematic showing the decomposition of misfit dislocations. (**a**) Stereographic pattern illustrating the decomposition of perfect Burgers vector a[0
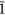
1] into two components of either parallel to or perpendicular to the line direction. (**b**) In-plane decomposition of a[0
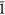
1] into two components of either parallel to or perpendicular to the line direction. Yellow arrow: perfect Burgers vector; blue arrow: edge component; red arrow: screw component; green arrow: direction of dislocation line.

Here, 1/2a[1
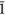
0] is the component that perpendicular to the direction of dislocation line. That is to say, this kind of dislocation is edge dislocation which should relax the lattice mismatch along (1
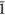
0) planes. 1/2a[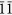
2] is the component which parallel to the direction of dislocation line. In contrast, this kind of dislocation is screw dislocation which could relax the shear strains in neighboring domains^25^ and the shear strains results from the anisotropic strains from the substrates. The schematic diagram showing the Burgers vector decomposition is illustrated in Fig. 6(a,b).

To generalize the misfit strain relaxation behavior in PTO/LAO (111) system, we have further prepared films with different thicknesses. Similar strain relax processes via a <110> misfit dislocations were persistently observed, which suggests the above mechanisms are common for PTO/LAO (111) system.

It is well known that if the mismatch is relatively small, films can grow coherently on substrates until a critical thickness is reached. When the film thickness exceeds the critical value, the introduction of misfit dislocations will be energetically preferred over fully coherent growth. For the <111> oriented perovskite films, the in-plane lattice constants d_110_ and d_112_ are very different. In addition, d_112_ and d_211_ are not equivalent as well. Therefore, the film bears anisotropic in-plane strains when grown along <111>. For perovskite film systems, it is generally believed that a misfit dislocation is generated either from the extension of pre-existing dislocations in the substrate or from the half-loop nucleation and expansion from the film surface by dislocation gliding or climbing and dislocation reaction^21^,^22^,^26^. So the process of misfit dislocation nucleation and multiplication is strongly dependent on the dislocation behavior in the films or substrates themselves. On one hand, if they were originated from the substrate, there should be high density of dislocations in LAO which terminated at the interface. But in our experimental, few dislocations in LAO substrate near the interface were observed. On the other hand, the critical thickness would be so small for large mismatch systems that it is not easy for dislocation half loops to form on the surface and then induce threading dislocations^22^. As a result, other factors may cause the formation of the misfit dislocations. It is known that the dominant driving force for the formation of misfit dislocations is strain energy, which is proportional to both the film thickness and the lattice misfit. So when the lattice misfit is large, misfit dislocations could nucleate at the interface during the beginning of film growth to reduce the huge strain energy. That is to say, this high density of misfit dislocations may directly form at the PTO/LAO interface simply during the deposition of PTO films. Langjahr *et al*.^22^ proposed similar suggestions for highly mismatched, [001] oriented perovskite systems. However, due to the anisotropy states of misfit strains on <111> oriented substrates and the tetragonal lattice of PTO, the distributions of misfit dislocations may be inhomogeneous. In addition, the polar discontinuities of the (111) surfaces may further facilitate the formation of a <110> type misfit dislocations during the growth of high index PTO films. What is more, if the misfit strain is small, the PTO film could be grown coherently. During the cooling step, the mismatch may be relaxed preferentially by the formation of ferroelectric domains rather than dislocations. In contrast, when the misfit strain is large like in this system, misfit dislocations will evolve first to effectively relieve the large mismatch during the deposition process. Nevertheless, the residual misfit strain could be relaxed by the formation of domains as evidenced by the domains denoted by blue arrow in Fig. 1b. The strain fields around misfit dislocation cores may also further trigger the formation of domains. Thus the final misfit dislocation configurations could be potentially affected by the interactions between the strain fields of dislocations and domains.

In summary, we have investigated the misfit strain relaxation behavior of (111)-oriented ferroelectric PTO epitaxial films grown on LAO (111). From the structural characterizations by conventional TEM and aberration-corrected TEM observations, we found that the growth of PTO (111) thin films is island-like Volmer-Weber mode due to the large misfit strains. The main approach for relaxing the misfit strains is the formation of misfit dislocations, which have mixed character with Burger vectors of a <110> and line directions of <112>. Only edge dislocation components relieve the misfit strains between the films and substrates. The screw dislocation components relax the shear strains resulting from ferroelectric domains and anisotropic strains from the substrate surfaces. The results reveal that the anisotropic and inhomogeneous in-plane strains coming from low symmetry substrate surfaces cause an unusual misfit relaxation behavior of the epitaxial PTO films. These findings may provide some insight for understanding the anisotropic strain relaxations in other (111)-oriented perovskite films, which demonstrate novel physical functionalities.

## Methods

### Sample preparation

Epitaxial thin films of PTO were grown on well-oriented LAO (111) substrates by PLD. A Lambda Physik LPX 305i KrF (λ = 248 nm) excimer laser was used. Before deposition, the substrate was heated at 750 °C for 20 min to clean the substrate surface and the laser was focused on a ceramic PTO target for 30 min pre-sputtering to clean the target surface. Throughout the deposition process, the substrate temperature was kept at 650 °C, with a laser energy density of 2 Jcm^−2^, a laser repetition rate of 4 Hz and under an oxygen pressure of 20 Pa. After deposition, the films were annealed at 650 °C in an oxygen pressure of 2 × 10^4 ^Pa for 60 min, and then cooled down to room temperature at a cooling rate of 5 °C min^−1^.

### Structural characterization

TEM specimens for cross-sectional observations were prepared by conventional method by cutting, gluing, grinding, dimpling, and finally ion-milling. A Gatan 656 Dimple Grinder was used for dimpling. Ar-ion-milling was performed by using a Gatan 695 Precision Ion Polishing System. Plane-view TEM samples were thinned and ion-milled only from the LAO substrate side until the Ar ion beam perforated the samples.

A Tecnai G2 F30 TEM working at 300 kV, was used for electron diffraction and diffraction contrast analysis.

Atomic resolution high-angle annular dark field (HAADF) STEM images in this study were recorded using aberration-corrected TEM (Titan^3TM^ G2 60–300 microscope fitted with a high-brightness field emission gun and a monochromator, and double Cs correctors from CEOS) operating at 300 kV. Strain fields were deduced by using custom plugins of geometrical phase analysis (GPA) for Gatan DigitalMicrograph^27^,^28^.

## Additional Information

**How to cite this article**: Xu, Y. B. *et al*. Misfit Strain Relaxation of Ferroelectric PbTiO_3_/LaAlO_3_ (111) Thin Film System. *Sci. Rep*. **6**, 35172; doi: 10.1038/srep35172 (2016).
